# The effect of informal caregiving on physical health among non-migrants and Ethnic German Immigrants in Germany: a cohort analysis based on the GSOEP 2000–2018

**DOI:** 10.1186/s12889-022-12550-0

**Published:** 2022-01-18

**Authors:** Daniela Georges

**Affiliations:** Faculty of Economic and Social Sciences, Institute of Sociology and Demography, University of Rostock, Ulmenstrasse 69, 18057 Rostock, Germany

**Keywords:** Health disparities, Longitudinal analysis, Long-term care, Immigrant health, Panel Analysis

## Abstract

**Background:**

The number of people in need of care in Germany has been rising since decades, which is related to an increasing need and relevance of informal caregiving. Likewise, the number of people with a migration background has been increasing. This study aims to analyse the impact of informal caregiving on physical health in comparative perspective for Ethnic German Immigrants (EGI) – the largest and oldest immigrant group in Germany – and non-migrant Germans (NMG).

**Methods:**

The sample was drawn from the years 2000–2018 of the German Socio-Economic Panel (*n* = 26,354). NMG (*n* = 24,634) and EGI (*n* = 1,720) were categorized into non-caregivers (*n* = 24,379) and caregivers (*n* = 1,975), where the latter were distinguished by 1) their caregiving status and history (current, former, and never caregiver) and 2) the number of years in the caregiver role. Generalized Estimating Equations were applied to examine main effects and the interaction effects of caregiving status and migration background for changes in physical health (*n* = 102,066 observations).

**Results:**

Adjusting for socioeconomic, household related, and individual characteristics, NMG and EGI had similar caregiving patterns and physical health. However, the interaction between migration background and caregiving revealed significantly higher declines in physical health for currently caregiving EGI. Sensitivity analyses indicated that particularly socioeconomic resources moderated this effect.

**Conclusions:**

Findings suggest that caregiving is associated with declines in physical health, particularly in the long term and for EGI. This implies that care-related disadvantages accumulate over time and that the association of caregiving, health and associated determinants are culturally diverse and shaped by migration background. Both the health disadvantages of caregivers and EGI might be mitigated by a positive social and socioeconomic setting, which highlights the relevance of supporting structures and benefits for these subgroups.

**Supplementary Information:**

The online version contains supplementary material available at 10.1186/s12889-022-12550-0.

## Background

Ageing societies and the associated increase in the number of people requiring long-term care (LTC) coincidently increase the number of individuals providing care for the spouse, for relatives, friends, neighbours, or other loved ones [1–3]. Providing such (usually unpaid) informal care can be demanding and research reports a negative impact of caregiving on the caregiver’s physical and psychological health [4–6]. Another current demographic development concerns the growing number of people with migrant status in many European countries [7, 8], who gradually will also reach care-relevant ages and will require LTC. This article analyses the impact of informal caregiving on physical health and its difference between migrants and non-migrants in Germany.

### LTC in Germany: demand, LTC insurance and supply

Approximately 14% of the population in Germany (~ 11.6 million persons) have lasting limitations in instrumental activities of daily living [9]. Of these, 4.1 million have a recognized need for LTC (“care level”). In Germany, a “care level” expresses the degree of need for care of a person, which is officially assessed and determined by the LTC insurance funds, and is linked to benefits from the statutory social care insurance [10]. Eligibility for benefits is based on an assessment of individual daily living abilities; it depends on the amount and intensity of support needed and includes care benefits (for inpatient or outpatient professional care) and/or care allowance (financial benefits if the care is provided informally). In contrast, the majority of people with limitations do not receive state benefits, i.e. irrespective of disability no care level has been requested or approved. Both groups usually live and are cared for at home, thus informal home care is a central pillar of the German care system [10, 11]. This informal care is provided by 9% of the adult German population [12], 61% of which within households, usually by close relatives, particularly by children (37%) or partners (32%) [13].

### Caregivers’ health

So far, the majority of studies on the impact of (informal) care on health has focussed on psychological health, while research on physical health is much less available [14, 15]. The impact of caregiving on physical health has been reported ambiguously in previous studies [16, 17], but tends to be negative [17–19] due to three mechanisms. First, there are health spillovers within families and household members, i.e. the illness – such as care need – of one member induces health decreases of the others [20, 21]. Second, transition into caregiving is accompanied by occupational, social, and organizational strains [22, 23]. And third, caregiving is physically demanding and induces physical stress [24]. Vitaliano et al. (2003) derived the caregiving-health-association as a path from the onset of caregiving through distress and physiological responses to illness. Psychological reactions therefore precede physical reactions [25]. This model has been proven in terms of psychological and physical health [26, 27], and path dependency [28]. The spillovers and the adverse effects of care were particularly pronounced in couples [27, 29].

Considering Stress and Coping Models [30, 31], the intensity and speed of caregiving effects on health are determined by needs and resources. “Needs” include both requirements of the care recipient, such as type/cause of care need, scope of limitations, amount of care need, and other obligations, such as employment or (further) family responsibilities, while “resources” cover economic, emotional, social, and personality characteristics. Higher needs and lower resources elevate the negative effects of care and enhance coping strategies. Thus, for example, long-term caregivers, persons with higher stress levels, carers with multiple caregiving roles, and ethnic minorities are more affected by caregiving [16, 32–34].

### Caregiving, health and ethnic differences

Culturally diverse aspects, such as family and role models, perceptions of illness, the acceptance of external care provisions, and motives to provide care, shape need-resource-patterns and the impact of caregiving on health [35]. Cultural differences are linked to different health care utilization patterns and behaviours in the case of care need [36, 37], and they have a complex effect on coping strategies [38]. Referring to Stress and Coping Models, ethnic differences in terms of socioeconomic status, family responsibilities, and individual resources must be assumed [39]. Additionally, temporal and situational differences, i.e. the timing of caregiving in the life course and the care patient’s characteristics, might contribute to ethnic differences [40]. Regarding physical health, studies have found greater health disadvantages among caregiving immigrants [35, 41, 42].

This study covered Ethnic German Immigrants (EGI), who are the oldest and largest group of persons with an immigrant background in Germany. In the year 2020, approximately 2.5 million EGI lived in Germany [43], with high levels of EGI immigration taking place in the 1990s [44]. EGI are descendants of people who emigrated from Germany before the 20^th^ Century or who stayed in former German regions after the Second World War. This means they are “Germans by status”, and they can acquire German citizenship directly [45]. Usually, EGI have migrated to Germany voluntarily and have unrestricted access to social welfare benefits and health services [46]. EGI have a greater cultural proximity to the autochthonous population, higher educational levels, lower return migration rates, and an older age structure than other immigrant groups [46]. However, EGI differ from the autochthonous population in Germany because they (or their descendants) have witnessed minority experiences abroad, have a history of migration including integration processes, have a lower socioeconomic status, live in rather traditional family structures, have more traditional attitudes, claim most health services less often, and are more likely to receive informal care [35, 46–48].

Thus, while there are differences in terms of needs and resources, no temporal and situational care-related differences between EGI and NMG have been identified thus far [49]. Hence, considering EGI are motivated by two gainful characteristics: firstly, they are currently one of the few immigrant populations that have already reached care-relevant ages. Considering that both EGI and other immigrant groups are united by the migration experience itself, findings for EGI might provide important insights for (yet) younger immigrant populations, e.g. Muslims or refugees. Secondly, the cultural, structural and legal proximity of EGI and NMG enables to examine the direct and indirect effects of migration background and minority status on the caregiving-health association, which is less influenced by further heterogeneity. EGI may undergo a higher stress level when becoming a caregiver due to different reasons, for example higher expectations to provide care informally within the family, lower rates of utilization of state and professional services, and lower social and economic resources. Therefore it was hypothesized that caregiving is more detrimental for the health of EGI than for NMG. It was further hypothesized that particularly in the long-term care is associated with worse health among EGI, when health-, care- and migration-related disadvantages accumulate.

## Methods

### Sample

**Fig. 1:**
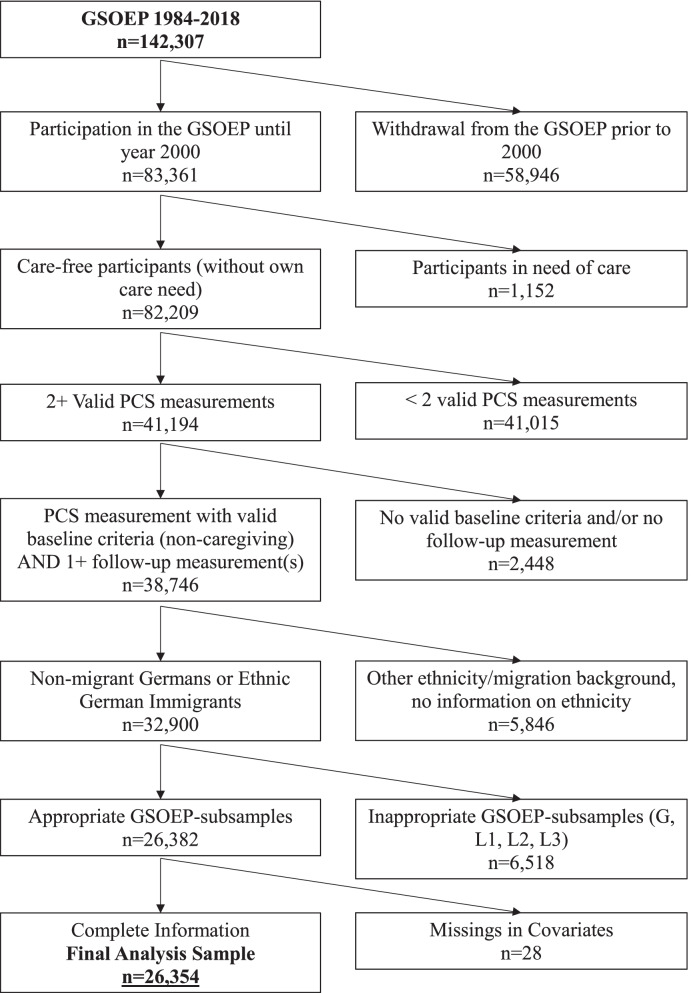
Sample Selection and Inclusion Criteria

This study used longitudinal data from the German Socio-Economic Panel covering the years 2000 to 2018 (GSOEP 2000–2018). The GSOEP is the largest and longest-running multidisciplinary, representative yearly household panel in Germany [50]. Due to its high annual response rates (e.g. 85.3% in 2018 [51]), and the relatively low panel attrition [52], the GSOEP was particularly suitable for this cohort study. A detailed health questionnaire has been included every two years since 2002. The years 2000 and 2001 were used to identify characteristics related to informal caregiving prior to the earliest possible baseline (in 2002) which are necessary to identify non-caregiving individuals in the year 2002. At baseline, the sample was restricted to NMG and EGI with at least one non-caregiving physical health measurement for a period of at least one year prior to the interview, i.e. in order to identify individuals with a valid baseline measurement in the year 2002, it was necessary to include information from the years 2000 and 2001. Excluding prevalent caregivers at baseline permits the study of the impact of the current caregiving status and the (observed) duration of caregiving. Moreover, individuals who have a need of care themselves were excluded to avoid interference. In the follow-up they needed to have at least one subsequent physical health measurement with or without caregiving. A large proportion of the observations of the data set from the years 2000 to 2018 had to be excluded due to an insufficient number of physical health measurements. Less than 2 physical health measurements were available for 41,015 individuals in the temporary data set (of which one measurement for 22,853 individuals, and no measurement at all for 18,162 individuals). This large proportion is driven by the fact that some of the individuals were surveyed for the last time prior to 2004 (7,796 individuals) respectively for the first time after 2016 (10,337 individuals), and thus per se could not achieve two health measurements. Panel attrition and mortality additionally contributed to a loss of analysable observations. The final analyses covered 26,354 individuals with a total of 102,066 observations of health changes. (see Fig. 1).

### Measures

#### Physical health changes

Physical health was measured using the Physical Component Summary (PCS), which is one of the two main dimensions of the Short-Form 12 Survey and subsumes six items: one on general health, one on physical pain, two on physical limitations, and two on physical health problems. PCS provided a metric scale from 0 to 100. Higher PCS scores indicated better health, where for each year the values were transformed and mean-centred to 50 (SD = 10) [53]. The observed outcomes were absolute changes in physical health from baseline onwards (Δpcs), calculated by ${\Delta pcs}_{j}$ = ${pcs}_{it+j}$ -${pcs}_{it=0}$, where ${pcs}_{it=0}$ denotes the physical health at baseline t = 0 and ${pcs}_{it+j}$ the physical health of each subject (i) in the next following valid year after baseline (t + j). Negative values indicated physical health deterioration and positive values health improvements since baseline. The number of health changes per subject (j) was between 1 and 8.

#### Caregiving

“Caregiving” referred to informal caregiving and was measured twofold: if either at least one person within the household mentioned being in need of care and/or if a person mentioned that s/he provided care for at least two hours on average weekdays. Both information referred to self-disclosure, whereby the granting of the need of care is based on a standardized medical assessment of the individual autonomy and functional limitations. The average duration of caregiving covered only subjective assessments and no information on the tasks associated with caregiving. All persons to whom at least one of these characteristics applied were categorised as “Caregivers”, all the other as “Non-Caregivers”. The incidence of the caregiver status was identified by a period of at least one non-caregiving year prior to the baseline. To analyse path dependencies, the group of caregivers is differentiated by the caregiving history and the current caregiving status from incident caregiving onwards (current caregivers, former caregivers). For the sensitivity analysis, the number of years in the caregiver role from incident caregiving onwards was included (metric and categorized into: 1–2 years; 3–4 years; 5 + years).

#### Migration background

Non-migrant Germans (NMG) and Ethnic German Immigrants (EGI) were provided two separate categories. All subjects for whom none of the characteristics included in the GSOEP (own/parental migrant history, country of birth, country of origin, nationality, immigrant group, and sample group) indicated an immigrant background were defined as NMG. The status of belonging to the immigrant group of EGI was asked directly (“Which of the following immigrant categories did you belong to when you moved to Germany?” […] “Person of German descent from Eastern Europe”) and was used as a classification criterion.

#### Covariates

**Fig. 2:**
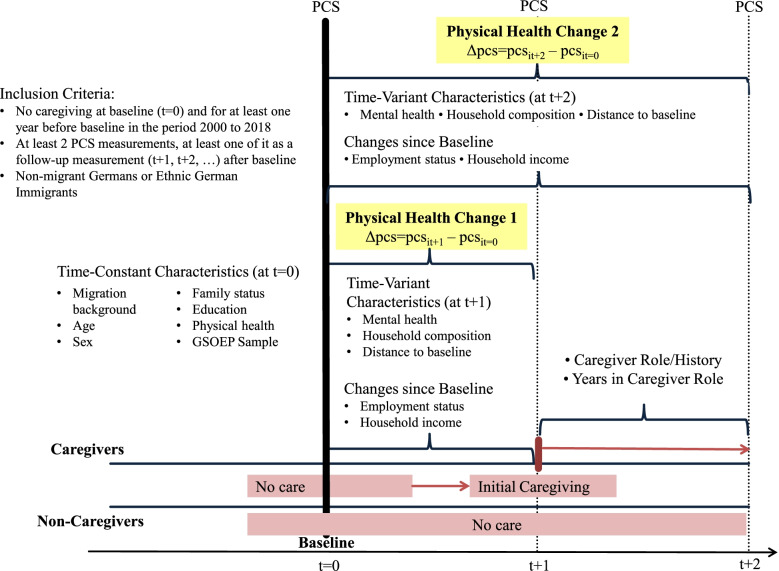
Framework, Study Design and Characteristics

The set of covariates covered socio-economic and household-related health determinants that particularly reflected the “resource” dimension of the abovementioned Stress and Coping Models and that have been identified as essential in earlier studies [6, 54], and included time-constant and time-variant characteristics. Time-constant characteristics referred to the baseline year and included age (< 50 years; 50–59; 60–69; 70–79; 80 +), sex (male; female), family status (unmarried; married-living together; married-not living together; divorced; widowed), education (based on the International Standard Classification of Education (ISCED) 1997: lower than middle vocational (i.e. in school, inadequately, general elementary (ISCED levels 0–2)); middle vocational (ISCED level 3); vocational + Abitur (ISCED level 4); higher vocational (ISCED level 5); higher (ISCED level 6)), and baseline physical health (PCS, metric, mean centred). Time-variant characteristics were changes from baseline onwards in employment status (status at baseline (full time; part time; marginally employed; non-working)*change in working hours (more; less; constant)) and household income (income quartiles at baseline*change (decrease (> -10%); increase (> + 10%); constant)), mental health (Mental Component Summary, metric, mean centred), and household composition (single household; couple household (without children); single parents; couple with underage children (< 16 years); couple with adult children (age 16 + years); multigenerational household; others/missing). In addition, two design variables were accounted for: the GSOEP-subsample (time-constant; at baseline), and the distance between follow-up PCS measurement and baseline (in years, metric, time-variant). The study design is illustrated in Fig. 2.

### Data analysis

Descriptive analyses were performed to report background characteristics of the sample and compositional differences by migration background and caregiving status. Bivariate analyses by chi-square tests and t-tests were performed to determine bivariate associations between caregiving status and covariates respectively migration background and covariates. Multivariate analyses for PCS changes were based on Generalized Estimating Equations (GEE) [55]. The models were specified with the identity link function for normally distributed outcomes, and with an independent within-person residual covariance matrix which proved to be the best covariance structure using the qic routine in Stata [56, 57]. Analyses comprised GEE models for the whole sample, and GEE models with interaction effects. Three step-wise models with subsequently added mediators and covariates were estimated: Model 1 included migration background and caregiving status, age, sex, baseline physical health and mental health as covariates, and accounts for the two design variables (distance to baseline; GSOEP-subsample). Household characteristics were added in Model 2, and socioeconomic characteristics were added in Model 3. Sensitivity analyses included tests for different thresholds of caregiving hours to distinguish caregivers and non-caregivers, analyses including the number of (observed) years a person provided care, models including care-related characteristics (i.e. information on the number of caregiving hours, the presence of a person in need of care within the household) for the total sample and the caregiver-subsample, models without adjustment for mental health, and models stratified by migration background.

## Results

### Descriptives

The sample comprised 102,066 observations, with an average physical health decline of -2.01 scale points. Of these observations, 6,992 (6.85%) referred to the period since initial caregiving and 5,254 (5.15%) were from EGI (Table 1).

**Table 1:** PCS and ΔPCS by Caregiving Status and Migration Background (*n* = 26,354; *N* = 102,066)

Groups (individuals/observations)	PCS at baseline (sd)	ΔPCS (sd)
Sample (*n* = 26,354/102,066)	51.24 (9.22)	-2.01 (8.62)
NMG (*n* = 24,634/96,812)	51.30 (9.19)	-2.03 (8.60)
EGI (*n* = 1,720/5,254)	50.14 (9.79)	-1.65 (8.89)
Non-Caregivers (*n* = 24,379/95,074)	51.43 (9.18)	-1.96 (8.55)
NMG (*n* = 22,723/90,136)	51.49 (9.15)	-1.98 (8.53)
EGI (*n* = 1,616/4,938)	50.36 (9.73)	-1.60 (8.86)
Caregivers (*n* = 1,975/6,992)	48.66 (9.40)	-2.63 (9.46)
Current caregivers (*n* = 1,881/2,724)	48.18 (9.47)	-2.38 (9.41)
NMG (*n* = 1,784/2,569)	48.27 (9.44)	-2.29 (9.35)
EGI (*n* = 97/155)	46.64 (9.86)	-3.89 (10.28)
Former caregivers (*n* = 1,495/4,268)	48.97 (9.35)	-2.78 (9.49)
NMG (*n* = 1,430/4,107)	49.07 (9.30)	-2.86 (9.53)
EGI (*n* = 65/161)	46.53 (10.23)	-0.91 (8.29)

Calculations based on GSOEP 2000–2018

All ‘Caregivers’ have at least one observation as ‘Current caregivers’ or as ‘Former caregivers’

Compared to non-caregivers, caregivers had higher physical health declines (-2.63 vs. -1.96) and lower physical health at baseline (48.66 vs. 51.43) (Table 1). Within the group of caregivers, physical health declines were remarkably lower for current caregivers than for former caregivers (-2.38 vs. -2.78). There were mixed results when comparing NMG and EGI. Non-caring EGI had lower physical health at baseline than NMG (50.36 vs. 51.49), but lower physical health declines over time (-1.60 vs. -1.98). Currently caregiving EGI had remarkably higher physical health declines (-3.89 vs. -2.29) and former caregiving EGI had remarkably lower physical health declines (-0.91 vs. -2.86) than did NMG counterparts. However, the lower physical health at baseline of caregiving EGI compared to caregiving NMG is noticeable (current caregivers: 46.64 vs. 48.27; former caregivers: 46.53 vs. 49.07) (Table 1).

In the sample, the majority was younger than 50 years (62.50%), female (52.06%), married and living together at baseline (58.28%), lived in a couple household without children (37.45%) or with (underage or adult) children (37.93%), had a middle vocational degree (52.14%), and worked full time at baseline (46.27%). While household income generally increased over the time period (47.04%), the employment status and the working hours did not change that much (no change: 70.40%). Both physical health at baseline (PCS = 51.24) and mental health (MCS = 50.67) were slightly above the population average.

Considering differences by migration background, EGI were more frequently never or currently caregiving, were slightly more frequently in the middle age groups (ages 50 to 79), were more frequently married and living together or living in couple households with children (particularly underage children), had slightly more frequently educational degrees up to “middle vocational”, had lower household incomes, and were more frequently working part-time or constantly non-working (each *p* < 0.05). While physical health at baseline and physical health declines over time were significantly lower for EGI, mental health (MCS) was significantly higher. The physical health change over time and rates of caregiving did not differ significantly between EGI and NMG. (Table 2; detailed results upon request).

**Table 2:** Characteristics of the Sample (*n* = 26,354), NMG (*n* = 24,924), and EGI (*n* = 1,430)

		**Total**		**NMG**		**EGI**		**Difference NMG—EGI**
Variable		N	%	N	%	N	%	*p*-value
	**Total**	**102,066**	**100**	**96,812**	**100**	**5,254**	**100**	
Physical Health at baseline (PCS) (mean) (t-c)		51.24		51.30		51.03		***a
Change (compared to baseline) (t-v) (mean)		-2.01		-2.03		-1.65		*a
Caregiving (t-v)	Non-Caregivers	95,074	93.15	90,136	93.10	4,938	93.99	***
	Former caregivers	4,268	4.18	4,107	4.24	161	3.06	
	Current caregivers	2,724	2.67	2,569	2.65	155	2.95	
Years in caregiver role (t-v)	0 (non-caregivers)	95,074	93.15	90,136	93.10	4,938	93.99	*
	1–2 years	4,928	4.83	4,719	4.87	209	3.98	
	3–4 years	1,222	1.20	1,159	1.20	63	1.20	
	5 + years	842	0.82	798	0.82	44	0.84	
Years in caregiver role (mean) (t-v)		0.09		0.09		0.09		a
Migration back-ground (t-c)	Non-migrant Germans (NMG)	96,812	94.85	96,812	100.00			
	Ethnic German Immigrants (EGI)	5,254	5.15			5,254	100.00	
Age (years) (baseline) (t-c)	< 50 years	63,787	62.50	60,584	62.58	3,203	60.96	*
	50–59	16,110	15.78	15,227	15.73	883	16.81	
	60–69	14,785	14.49	14,018	14.48	767	14.60	
	70–79	6,338	6.21	5,982	6.18	356	6.78	
	80 +	1,046	1.02	1,001	1.03	45	0.86	
Age (years) (baseline) (mean) (t-c)		44.23		44.21		44.48		a
Sex (t-c)	Male	48,933	47.94	46,560	48.09	2,373	45.17	***
	Female	53,133	52.06	50,252	51.91	2,881	54.83	
Mental Health (MCS Scale) (mean) (t-v)		50.67		50.65		51.03		**a
Family status (baseline) (t-c)	Unmarried	28,233	27.66	27,293	28.19	940	17.89	***
	Married—living together	59,489	58.28	55,755	57.59	3,734	71.07	
	Divorced	7,681	7.53	7,381	7.62	300	5.71	
	Widowed	4,771	4.67	4,574	4.72	197	3.75	
	Married—not living together	1,892	1.85	1,809	1.87	83	1.58	
Household composition (t-v)	Single household	17,952	17.59	17,256	17.82	696	13.25	***
	Couple household (w.o. children)	38,222	37.45	36,388	37.59	1,834	34.91	
	Single parents	5,507	5.40	5,229	5.40	278	5.29	
	Couple w underage children	23,314	22.84	21,783	22.50	1,531	29.14	
	Couples w adult ch. (age 16 +)	15,404	15.09	14,603	15.08	801	15.25	
	Multigenerational household	855	0.84	791	0.82	64	1.22	
	Other composition/missing	812	0.80	762	0.79	50	0.95	
Education (t-c)	Lower than middle vocational	9,267	9.08	8,448	8.73	819	15.59	***
	Middle vocational	53,218	52.14	50,874	52.55	2,344	44.61	
	Vocational + Abitur	6,420	6.29	5,676	5.86	744	14.16	
	Higher vocational	8,551	8.38	8,175	8.44	376	7.16	
	Higher	24,610	24.11	23,639	24.42	971	18.48	
Household Income at baseline (quartiles Q1-Q4) * Change (t-v)	Q1., Decrease > -10%	4,713	4.62	4,392	4.54	321	6.11	***
	Q2., Decrease > -10%	6,889	6.75	6,447	6.66	442	8.41	
	Q3, Decrease > -10%	8,043	7.88	7,661	7.91	382	7.27	
	Q4, Decrease > -10%	8,139	7.97	7,911	8.17	228	4.34	
	Q1, Constant (± 10%)	7,108	6.96	6,659	6.88	449	8.55	
	Q2, Constant (± 10%)	7,432	7.28	6,992	7.22	440	8.37	
	Q3, Constant (± 10%)	7,212	7.07	6,870	7.10	342	6.51	
	Q4, Constant (± 10%)	4,518	4.43	4,392	4.54	126	2.40	
	Q1, Increase > + 10%	16,377	16.05	15,329	15.83	1,048	19.95	
	Q2, Increase > + 10%	13,792	13.51	12,935	13.36	857	16.31	
	Q3, Increase > + 10%	11,718	11.48	11,203	11.57	515	9.80	
	Q4, Increase > + 10%	6,125	6.00	6,021	6.22	104	1.98	
Employment Status at baseline * Change (t-v)	Full time—No Change	35,327	34.61	33,631	34.74	1,696	32.28	***
	Full time—Reduced	11,898	11.66	11,384	11.76	514	9.78	
	Part time—More	1,727	1.69	1,624	1.68	103	1.96	
	Part time—No Change	5,913	5.79	5,576	5.76	337	6.41	
	Part time—Reduced	3,062	3.00	2,914	3.01	148	2.82	
	Non-regular—More	1,828	1.79	1,729	1.79	99	1.88	
	Non-regular—No change	1,632	1.60	1,283	1.33	79	1.50	
	Non-regular—Reduced	1,382	1.35	1,320	1.36	62	1.18	
	Non-working—More	10,581	10.37	9,962	10.29	619	11.78	
	Non-working—No change	28,986	28.40	27,389	28.29	1,597	30.40	

Calculations based on GSOEP 2000–2018

*p*-value for categorical variables for difference between NMG and EGI is based on a chi-square test between migrant group and the independent variable, *p*-value for metric variables (a) is based on a t-test, *t-v* time-varying variables, *t-c* time-constant variables, *p < .05, **p < 0.01, ***p < 0.001

Considering differences by caregiving status, physical health at baseline was highest among non-caregivers and differed significantly across the groups. Physical health declines over time were significantly lower for non-caregivers than for caregivers but did not differ significantly within the group of caregivers. Moreover, caregivers were more frequently females, had more frequently low or medium education levels, more frequently reduced the number of hours worked, and were older than non-caregivers. The latter was particularly true for current caregivers, who moreover were more frequently married and lived without children. Finally, a decrease of the household income was most frequently among former caregivers. (see Additional File 1).

### GEE models

GEE models were estimated to adjust for additional covariates and to adjust for structural differences by migration background and caregiving status as abovementioned described. Model 1 was the basic model, and included migration background and caregiving status as the main explanatory variables, and some covariates. Caregiving and migration background significantly affected physical health changes (Table 3, Model 1). Caregivers had significantly higher physical health declines, which were more pronounced for current caregivers (-0.73, 95% CI: -1.02,-0.44) than for former caregivers (-0.54, 95% CI: -0.78, -0.30). Additionally, among EGI health declined significantly faster (-0.56, 95% CI: -0.79, -0.34) than among NMG (Additional File 2, Model 1). Adjusting for household characteristics in Model 2, care-related health disadvantages (former caregivers: -0.45, 95% CI: -0.69, -0.22; current caregivers: -0.63, 95% CI: -0.92, -0.34) were reduced, but remained significant, and differences by migration background were almost the same (EGI: -0.55; 95% CI: -0.77, -0.32) (Table 3, Model 2; Additional File 2, Model 2). Socioeconomic characteristics, included in Model 3, largely mediated the main effects. Both, the health of former caregivers (-0.32; 95% CI: -0.55, -0.09) and the physical health of current caregivers (-0.44; 95% CI: -0.72, -0.15) deteriorated significantly faster than that of non-caregivers. Moreover, among EGI physical health declines were significantly more pronounced than for NMG (-0.32; 95% CI: -0.55, -0.10) (Table 3, Model 2; Additional File 2, Model 2). Regarding the control variables, applying the Wald test, it can be shown that (apart from sex) each characteristic was associated with physical health changes (each *p* < 0.01) (Table 3, Model 3). Adjusted for migration background, caregiving, and the respective other covariates, physical health declined faster at older ages, among divorced, widowed or separately living married individuals, for all household compositions compared to couples with underage children, for persons with non-middle vocational or higher vocational education levels, for those with income decrease and the first three income quartiles at baseline, and for those who reduced their working hours or were constantly non-working. Physical health declines were additionally higher for individuals with better physical health at baseline and with higher mental health (Additional File 2, Model 3).

**Table 3:** Linear Regression (GEE Models): Determinants of Physical Health Changes (overall *p*-values)

	**Model 1**	**Model 2**	**Model 3**
Variable	*p*	*p*	*p*
Caregiving (t-v)	< 0.001	< 0.001	< 0.001
Migration background (t-c)	< 0.001	< 0.001	0.005
Age (years) (baseline) (t-c)	< 0.001	< 0.001	< 0.001
Sex (t-c)	< 0.001	< 0.001	0.141
Physical Health at baseline (PCS) (mean) (t-c)	< 0.001	< 0.001	< 0.001
Mental Health (MCS Scale) (mean) (t-v)	< 0.001	< 0.001	< 0.001
Family status (baseline) (t-c)		< 0.001	< 0.001
Household composition (t-v)		< 0.001	< 0.001
Education (t-c)			< 0.001
Household Income at baseline (quartiles Q1-Q4) * Change (t-v)			< 0.001
Employment Status at baseline * Change (t-v)			< 0.001
Cons	< 0.001	< 0.001	< 0.001
N (obs.)	26,354	26,354	26,354
N (individuals)	102,066	102,066	102,066

Calculations based on GSOEP 2000–2018

Overall *p*-value for categorical variables based on Wald test; overall *p*-value for metric variables based on linear regression; all models were adjusted for design variables (distance to baseline, GSOEP-subsample), *t-v* time-varying variables, *t-c* time-constant variables, *NMG* Non-migrant Germans, *EGI* Ethnic German Immigrants

### Interaction effects between caregiving and migration background

**Fig. 3:**
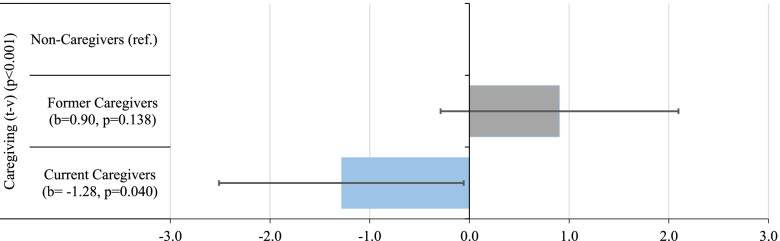
Interaction Effect Caregiving*Migration Background. Calculations based on GSOEP 2000–2018. (*N* = 102,066). The blue-coded bar indicates a significant different coefficient for EGI compared to NMG; overall *p*-value for the interaction effect based on Wald test; adjusted for all covariates

To examine migration-related mechanisms and differences in physical health changes and the association of caregiving and physical health changes, interaction effects were estimated. Again, the strategy was to apply three step-wise models, and subsequently add covariates. Overall, the interaction effect was significantly associated with physical health changes in all Models (*p* < 0.001), and thus, there were differences in the caregiving-health-association by migration background (Table 4; Fig. 3). Currently caregiving EGI had additional declines in physical health, and these significantly exceeded the declines of NMG (-1.28, 95% CI: -2.51, -0.06) (Table 4, Model 3). This tendency persisted across the three models, but was only significant adjusting for socio-economic characteristics.

**Table 4:** Interaction effects Caregiving*Migration Background (*n* = 26,354; *N* = 102,066) (overall *p*-values, coefficients and corresponding *p*-values)

		**Model 1**			**Model 2**			**Model 3**		
EGI		Coeff	95% CI	*p*	Coeff	95% CI	*p*	Coeff	95% CI	*p*
Care-giving (t-v)	Overall^a^			< 0.001		< 0.001				< 0.001
	Non-caregivers (ref.)	0			0			0		
	Former caregivers	1.09	[-0.12,2.30]	0.077	1.23	[0.03,2.43]	0.044	0.90	[-0.29,2.10]	0.138
	Current caregivers	-1.12	[-2.37,0.12]	0.076	-1.13	[-2.37,0.10]	0.072	-1.28	[-2.51,-0.06]	0.040

Calculations based on GSOEP 2000–2018

^a^Overall *p*-value for the interaction effect based on Wald test; coefficients represent additional differences compared to NMG counterparts; M1 adjusted for migration background, caregiving status, age, sex, baseline physical health, mental health, distance to baseline, GSOEP-subsample, M2 additionally adjusted for family status at baseline, household composition, M3 additionally adjusted for education, household income at baseline*change, employment status at baseline*change, *t-v* time-varying variable, *ref.* reference category

### Sensitivity analyses

Several sensitivity analyses were performed to evaluate the robustness of the results and potential third variables. Firstly, the main explanatory variable – caregiving – was checked. Caregivers were defined as all those who lived in a shared household with at least one person in need of care or who stated to care for others for at least two hours per day. This definition came close to definitions of previous studies [58, 59]. The results are largely robust, where lower thresholds predictably lead to slightly less distinct results, and higher thresholds are accompanied by higher statistically uncertainty due to the lower number of cases (20% of the caregiving observations referred to caregivers with two caregiving hours per day). Secondly, to model the care history more detailed, the number of years in the caregiver role since initial caregiving was used as the main explanatory variable (besides migration background). There is a negative association between the number of years and physical health (metric: -0.09, 95% CI: -0.15, -0.03; categorized: 0 years (ref.): 0; 1–2 years: -0.31, 95% CI: -0.53, -0.08; 3–4 years: -0.47, 95% CI: -0.90, -0.03; 5 + years: -0.52, 95% CI: -1.05, 0.00). Interacting with migration background, there were tendencies of worse physical health with increasing length of care among EGI, but these did not reach significance (each *p* < 0.10). Thirdly, to consider the burden associated with caregiving, a set of care-related characteristics was integrated. Both the number of daily caregiving hours and a shared household with a person in need of care (*N* = 1,214) had no distinct effect on physical health and did not interact with migration background. These characteristics were additionally tested on the caregiver-subsample (*N* = 6,992), but were not correlated with physical health and migration background. Additional characteristics related to the care recipient (care level, diseases, type of care need, external support) were considered and examined exploratory, but could not be included meaningfully, and did not deliver reliable results due to many missing information. Fourthly, to recognize the interrelation between physical and mental health (MCS), models without MCS were estimated. This slightly reduced the physical health differences depending on the caregiving status, but had almost no effect on the association and interaction between migration background and physical health. As MCS improved the model substantially, this variable has been maintained. Finally, to evaluate migration-specific mechanisms, models stratified by migration background were estimated. Apart from economic characteristics and family status – family status at baseline, education, income, and occupational status/changes were largely irrelevant for EGI – the mechanisms were very similar for both groups. All analyses are available on request.

## Discussion

In line with previous studies [17, 18] this study suggests physical health disadvantages among caregivers. These do both evolve directly with transition into caregiving and have a long-term effect beyond the care period. Although the impact of direct and indirect health spillovers [20, 21] could not be disentangled in this study, they might partly explain physical health disadvantages of caregivers. In addition, the occupational and social strains associated with incident caregiving [22, 23] seem to play a central role, as these characteristics moderated the health-care-relationship. Socioeconomic, household, and individual characteristics partially mediated the effect caregiving has on physical health, whereas older ages, lower levels of education, low incomes and income decreases, unemployment and leaving full-time occupation – and thus particularly socioeconomic characteristics – were associated with physical health declines. These findings are comparable to previous findings [27] and elucidate both the impact of economic changes associated with incidence of care [22, 23] and the mitigating effect of higher resources among caregivers [30]. Thus, interventions for the labour situation, e.g. a better reconciliation of care and employment, might reduce public health burdens and the health burden of caregivers [60, 61]. In addition, this study implies caregivers to have additional physical health disadvantages with increasing care duration over the life course [62]. This illustrates the accumulating negative impact caregiving has on physical health over time, and emphasizes the need to support caregivers in the long-term.

Differences in physical health changes between NMG and EGI were found across all models, indicating slightly higher health declines among EGI. Moreover, the results clarify that cultural differences shape the effect caregiving has on physical health [34], as the negative impact of caregiving is partly stronger among EGI. Currently caregiving EGI had additional physical health disadvantages over NMG by -1.28 points, which roughly equals the average (adjusted) difference between the age groups 60–69 and 70–79. Compared to the other coefficients, this was a noticeably strong effect. Neither socioeconomic, individual or household characteristics nor economic or employment changes explained these differences. However, socioeconomic resources were less important among EGI, while household characteristics appear to be more relevant and partly compensate for lower resources. The significant physical health advantages of caregiving EGI living in a couple household with underage children or living in a multigenerational household (results upon request) might indicate stronger intra-familial and intergenerational cohesion among EGI, which might reduce caregiver burdens [42]. Moreover, the transition into caregiving was culturally shaped. While the share of caregivers was similar for NMG and EGI, particularly EGI with poorer physical health at baseline transitioned into the caregiving role. However, while processing the data, it was noticeable that particularly EGI left the GSOEP after incidence of caregiving, thus their share was underestimated.

This study included main determinants of health, but could not fully cover the complex relationship between caregiving, physical health, and migration background. Thus, additional background characteristics and unobserved heterogeneity might be discussed. Considering stress and coping models, these cover sociocultural, interpersonal, and patient-related characteristics [32, 40]. While economic, organizational and psychological strains were integrated into the analyses, emotional and social strains [22, 63], motives to provide care [41, 42, 64], burdens associated with caregiving [65, 66], and external resources were not integrated depletive in favour of migration background and due to data restrictions. There might be differences by migration background in these characteristics. However, it is hypothesized that these only marginally mediate the strong and direct impact daily caregiving has on health. Subsequent studies might shed more light on characteristics of the care-recipient as well as the “need” dimension of caregiving considering stress and coping models. Path models could enable to understand passages and transitions, for example the transition into caregiving or the termination of caregiving.

As the analyses were based on longitudinal survey data, panel mortality and non-response might have biased the results. The share of persons with caregiving in the sample is 7.5% and thus slightly lower than other studies of Germany [12], but because only some years (up to a maximum timespan of 16 years per person) were analysed, this share seems reasonable. Nevertheless, the inclusion criteria might contribute to biased estimations. The sample only included non-caring individuals at baseline and thus prevalent as well as long-term caregivers were underrepresented. However, the sample’s average physical and mental health was slightly higher, but essentially equal to the health status of the population in Germany. Regarding selective panel mortality, it is likely that ill people, persons with burdensome family events, and migrants were less included the sample [67, 68]. Moreover, survey participation usually is not evenly distributed within the group of caregivers [69], and it has to be assumed that caregivers were underrepresented and positively selected, and thus the effects are rather underestimated. It must also be borne in mind that the information on the care status (depicting the presence of persons in need of care within the household and average care hours per working day) were based on unvalidated subjective self-assessment. The study design is associated with left- and right-censoring and thus with incomplete information, i.e. persons that used to provide care in the past might be misclassified as non-caregivers, while others were possibly not interviewed until transition into caregiving. Different selection into the sample by migration background cannot be completely excluded. Population-based surveys might solve this problem. Concerning selectivity into caregiving there is positive and negative selection, as persons living with underage children and non-higher vocational education levels are less likely to become caregivers, while persons aged 50 to 59, females, and individuals with better physical and mental health were more likely to provide care. Finally, EGI had a higher likelihood to turn into caregivers than NMG (*p* < 0.001; results upon request).

The longitudinal analyses based on Generalized Estimating Equations allowed for analysis of clustered data and determining associations of cause and effect in the limited framework of non-experimental studies. Background information was taken from the baseline year, which was measured before physical health changes by definition. Additionally, initial caregiving and information on the caregiving history always preceded the follow-up physical health measurement, which was used to quantify health changes. Interdependencies due to autocorrelation of time-variant characteristics, such as mental health, cannot be excluded, but GEE models consider these dependencies and are even robust in the case of misspecification [56, 70]. Usually, GEE models are highly eligible to perform epidemiological studies and cohort studies, and enable to measure average effects over the population of correlated data [71].

Despite the restrictions, the results indicate accumulating disadvantages of caregiving. Caregiving is emotionally and physically demanding and interventions to reduce the health risks of caregivers are needed to prevent caregivers from being the patients of tomorrow, and to reduce possible burdens on care systems and health systems. On the one hand, the analyses illustrate the breadth of potential mechanisms to reduce caregiver burdens, e.g. higher household incomes and employment. The positive effects of marriage and living with underage children underscore the relevance of social support and family cohesion. On the other hand, these results can only partly explain the correlation of caregiving and physical health. In addition to the aforementioned discussed characteristics, there may perhaps also be macrolevel factors [19] which determine the impact of informal caregiving and differences by migration background. Consultation and supporting structures, additional to benefits of the statutory long-term care insurance, might be helpful. The care-related physical health disadvantages of EGI, who are less likely to utilize state benefits and offers [72], emphasize the need to establish user-oriented and accessible state benefits. Moreover, considering these differences reveals the importance of multicomponent interventions at several levels to counteract caregiver burdens [65, 66].

Against the background of cultural and structural proximity of EGI and NMG, it may be discussed whether the associations might be even stronger for other immigrant groups [39]. In this study, in favor of homogeneity no additional groups were integrated. However, considering the care disadvantages of EGI, who are better integrated and healthier than other immigrant groups, it is plausible that the negative impact of caregiving might be even more pronounced for other immigrant groups.

## Conclusions

This study contributes to a better understanding of the mechanisms of the caregiving-health-association, and thus helps us to understand the interrelation of the ageing process and providing informal care. Current demographic developments are accompanied by an increasing number of people in need of care who have a migration background, and caregivers, particularly immigrant caregivers, are vulnerable groups. The use of most current data helps us to understand contemporary and future challenges, and emphasizes that a steady care situation might be related to additional challenges for public health in the short-, medium- and long-term. The integration of migration background allows a better understanding of cultural, social, and household-related differences. However, subsequent studies might integrate additional contextual effects and expand the long-term perspective to disentangle the main drivers of care burdens and to understand developments and interdependencies properly.

## Supplementary Information

**Additional file 1: **Characteristics of the Sample (*n*=26,354), Non-Caregivers (24,379), Current Caregivers (*n*=1,881), and Former Caregivers (*n*=1,495).

**Additional file 2: **Linear Regression (GEE Models): Determinants of Physical Health Changes (coefficients and corresponding *p*-values).

## Data Availability

The raw data were drawn from the German Socio-Economic Panel Study (version 35, https://doi.org/10.5684/soep.v35). The data which support the findings of this study are available from the German Institute for Economic Research (DIW), but restrictions apply to the availability of these data, which were used under license for the current study, and so are not publicly available. However, data are available from the authors for scientific research. All information about the public availability of the GSOEP data can be found at: https://www.diw.de/en/diw_01.c.601584.en/data_access.html.

## Abbreviations

GSOEP: German Socio-Economic Panel
EGI: Ethnic German Immigrants
NMG: Non-migrant Germans
PCS: Physical Component Summary
SD: Standard Deviation
GEE: Generalized Estimating Equation
MCS: Mental Component Summary
T-c: Time-constant
T-v: Time-varying
Ref.: Reference category
95% CI: 95% Confidence Interval
ISCED: International Standard Classification of Education
